# Macular development in preterm twins with and without retinopathy of prematurity: an OCT angiography study from the Shenzhen prematurity eye study

**DOI:** 10.1186/s40001-026-03848-5

**Published:** 2026-01-28

**Authors:** Honglang Zhang, Xinyu Zhao, Zhenquan Wu, Kaixuan Cui, Zhen Yu, Yaling Liu, Cui Wang, Guoming Zhang

**Affiliations:** 1 The Second Clinical Medical College of Jinan University, Shenzhen Eye Hospital, Shenzhen, 518040 China; 2 Shenzhen Eye Hospital, Shenzhen Eye Medical Center, Southern Medical University, 18 Zetian Road, Futian District, Shenzhen, 518040 China

**Keywords:** Twins, Retinopathy of prematurity, Optical coherence tomography angiography, Visual acuity

## Abstract

**Purpose:**

To evaluate the macular microvascular and structural characteristics in preterm twins aged 3 to 12 years using optical coherence tomography angiography (OCTA), and to investigate the impact of perinatal factors, including gestational age (GA), birth weight (BW), and birth order, on macular development and visual function.

**Methods:**

This retrospective cohort study included 50 preterm twins (25 pairs, 98 eyes) followed at Shenzhen Eye Hospital from 2018 to 2023. All participants underwent OCTA and visual acuity examination. OCTA parameters and best-corrected visual acuity (BCVA) were compared between groups stratified by BW and birth order. Generalized estimating equation (GEE) models were used for statistical analysis. Univariable and multivariable analyses were used for retinopathy of prematurity (ROP) risk factor analyses and for association analyses of perinatal and OCTA parameters with BCVA and foveal avascular zone (FAZ) area.

**Results:**

ROP was diagnosed in 24 of 50 preterm twins (48%). No significant differences were found in OCTA parameters and BCVA between two BW groups and between birth-order groups. In multivariable analysis, cesarean section was associated with a lower risk of ROP than vaginal delivery (OR = 0.049, P = 0.005). Lower GA was independently associated with worse BCVA (P = 0.03) and a smaller FAZ area (P < 0.01).

**Conclusion:**

BW and birth order had no significant effects on macular or visual development. Lower GA was associated with smaller FAZ area and poorer visual acuity in preterm twins.

## Introduction

Prematurity is a major risk factor for childhood visual impairment and is associated with retinopathy of prematurity (ROP), refractive errors, and strabismus [1–3]. Previous studies suggest that prematurity may disrupt macular vascularization and foveal microstructural maturation, including disorganization or persistence of inner retinal layers [4–6]. Among perinatal factors, lower birth weight (BW) and gestational age (GA) have been associated with delayed retinal and macular development [7, 8] and these alterations may contribute to later visual dysfunction [9–11]. Therefore, objective and long-term evaluation of macular development and visual function is clinically relevant for surveillance in children born preterm.

Twin pregnancy is associated with a higher risk of preterm birth. Population-based data from developed countries suggest that approximately 60% of twins are born preterm (< 37 weeks) [12]. By comparison, the World Health Organization has reported a global preterm birth rate of 10.6% [13]. For preterm twins, most long-term research has focused on neurological development [14, 15] and genetic influences [16, 17]. Long-term ophthalmic evidence remains limited, particularly for school-age macular microvascular and structural outcomes. Twin-pair designs have also been used infrequently to test whether within-pair differences, such as BW discordance or birth order, are associated with later macular development and visual function. This evidence gap limits risk stratification and long-term follow-up strategies for preterm twins.

Optical coherence tomography angiography (OCTA) revolutionizes retinal imaging through its capacity for non-invasive, high-resolution, visualization of the retinal microvasculature without exogenous dye administration, offering a safer and more efficient alternative to traditional angiography methods [18–20]. OCTA allows quantitative assessment of the foveal avascular zone (FAZ) and perfusion-related metrics such as perfusion density (PD) and vessel length density (VLD) [21], facilitating objective characterization of macular microvasculature in school-age children with a history of prematurity.

This study used OCTA to characterize long-term macular microvascular and structural features and best-corrected visual acuity (BCVA) in preterm twins aged 3–12 years and to evaluate associations with perinatal factors, thereby helping to inform long-term risk stratification and follow-up strategies in this population.

## Methods

This study follows the principles outlined in the Helsinki Declaration and obtained informed consent from the parents before ophthalmic examination. The study was approved by the Institutional Review Board of Shenzhen Eye Hospital (IRB No: 2025KYYJ100-01). All data were anonymized prior to analysis.

### Study design and population

This retrospective study employs OCTA (software v9.5, Carl Zeiss Meditec, Dublin, California, USA) to assess retinal structure and vascular characteristics in twin children. All data were obtained from our Eyecare-cloud platform [22], a comprehensive pediatric ophthalmology database aggregating over 40 thousand cases from many hospitals across China, including over two thousand premature infants followed over two decades. This database previously supported our OCTA research in children with history of ROP [23], where we demonstrated significantly lower central VLD and PD in ranibizumab-treated versus laser-treated eyes, providing the methodological foundation for this twin-cohort analysis.

**Fig. 1:**
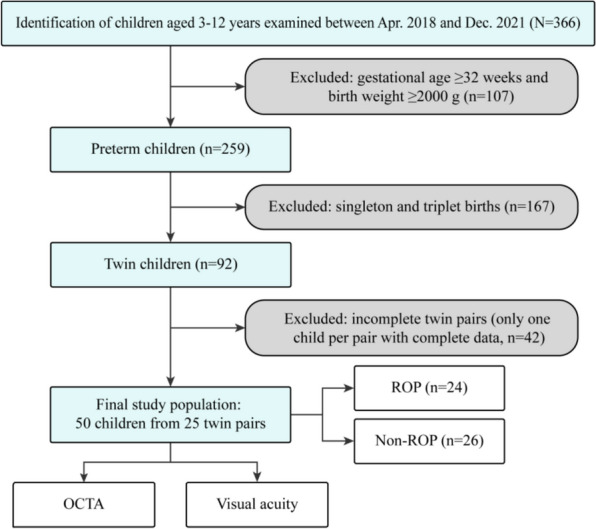
Flowchart of participant inclusion. Among 366 children who underwent OCTA examination, exclusions resulted in 50 children from 25 complete twin pairs (ROP: 24; non-ROP: 26) who completed both OCTA and visual acuity examination. OCTA, optical coherence tomography angiography; ROP, retinopathy of prematurity

The study design flowchart is presented in Fig. 1. A total of 366 children aged 3–12 years who underwent OCTA and visual acuity examinations between April 2018 and December 2023 were initially identified at Shenzhen Eye Hospital. Based on the ROP screening criteria in China [24], 107 children with gestational age ≥ 32 weeks or birth weight ≥ 2000 g were excluded. Additionally, 167 singleton or triplet births were excluded. To ensure complete paired data for twin analysis, 42 children from incomplete twin pairs (where only one child had complete data) were further excluded. The final study population comprised 50 preterm children from 25 twin pairs. This cohort was subdivided into two groups: 24 children with a confirmed history of ROP, and 26 without. ROP diagnosis was obtained from the medical records. All ROP diagnoses were further reviewed and confirmed by two pediatric retinal specialists with more than 10 years of clinical experience, based on the standard clinical documentation in the record (including stage and zone when available). In addition, we excluded participants with documented congenital ocular abnormalities or other ocular diseases that could affect OCTA measurements or visual acuity. Finally, OCTA images were unavailable for two eyes from two children (one eye per child) because of poor image quality (Q < 7); these eyes were excluded from analysis, while the children and their fellow eyes were retained. Consequently, a total of 98 eyes were included in the final analysis.

Twin children were classified into high BW and low BW groups using a within-pair comparison. For each twin pair, the infant with the higher birth weight was assigned to the high BW group and the co-twin with the lower birth weight to the low BW group, irrespective of absolute BW cut-off values. Subsequently, twins were further stratified by birth order into first-born and second-born groups. Birth order was defined according to delivery records in our database.

### OCTA image acquisition and analysis

All children underwent OCTA imaging using the Cirrus AngioPlex 5000 system with a 3 × 3 mm scanning mode. The superficial retinal layer was captured from the internal limiting membrane to the inner plexiform layer. OCTA image quality was assessed using the device’s built-in signal strength (Q) index. Only images with a Q score ≥ 7 were included in the analysis. Scans with motion artifacts, segmentation errors involving the inner retinal boundary, defocus, or media opacity–related shadowing in the central 3 × 3 mm area were excluded. Image quality was evaluated on-screen by the same certified ophthalmic technician at the time of acquisition, and questionable scans were further reviewed by a senior ophthalmologist.

FAZ is a central region of the fovea devoid of capillaries. PD is defined as the percentage of the area occupied by the vessels in a particular area, and it is shown as mm^2^/mm^2^. VLD is defined as the sum of the length of vessels in a particular area, and it is shown as mm/mm^2^ [25]. The foveal zone was defined as follows: the central fovea was delineated as 1 mm diameter circular zone, the foveal region as 3 mm diameter circular zone, and the parafoveal area as 2 mm wide annular region surrounding the central fovea. The parafoveal area was further subdivided into four quadrants of 90 degrees each: nasal, temporal, superior, and inferior quadrants for detailed regional analysis. The total parameters using OCTA were as follows: central foveal VLD and PD, parafoveal VLD and PD, foveal the VLD and PD, FAZ area, FAZ boundary and FAZ circularity, and parafoveal VLD and PD for each of the four quadrants (nasal, temporal, superior, and inferior). Central foveal thickness (CFT) was measured from internal limiting membrane to retinal pigment epithelium’s inner border [26]. The OCTA data (e.g., VLD, PD, FAZ) and CFT were measured by the software automatically. To ensure consistency, all examinations were conducted by a single certified ophthalmic technician using the same imaging system under standardized conditions.

### Cycloplegic refraction

Examinations were performed in a fixed order: cycloplegic refraction was conducted first, followed by OCTA imaging in the same visit. After cycloplegia was induced for refraction (0.5% tropicamide, four doses at 5-min intervals), pupillary dilation was verified when achieving ≥ 6 mm diameter. Refractive assessment was then performed by an experienced optometrist in a dark room. BCVA was determined using a back-illuminated Snellen chart at 5-m distance, with results converted to logarithm of minimal angle of resolution (LogMAR) units for quantitative analysis. In total, BCVA data were missing for 12 children aged 3–5 years due to poor cooperation; these children were excluded from BCVA analyses, whereas cycloplegic refraction (sphere and cylinder) was still successfully obtained for all participants. In accordance with the International Myopia Institute classification system for pediatric refractive errors [27], we categorized all participants into myopia (spherical equivalent [SE] ≤ − 0.50 diopters) and non-myopia (SE > − 0.50 diopters) groups, with SE calculated as SE = sphere + (cylinder/2).

### Statistical analysis

Statistical analysis was performed using SPSS v26.0 software (IBM Corp., Armonk, NY, USA) and the R 4.5.1 (R Core Team, Vienna, Austria). Continuous variables were expressed as mean ± standard deviation, while categorical variables were expressed as frequencies. To compare OCTA parameters and visual acuity between groups (high vs. low BW; first-born vs. second-born), generalized estimating equations (GEE) were used to account for the correlation between both eyes of the same subject. Independent samples t-tests or Mann–Whitney U tests were applied for continuous variables, and χ^2^ test or Fisher’s exact test for categorical variables.

To evaluate associations between variables, univariable and multivariable analyses were performed using GEE models to account for bilateral ocular parameters and associated risk factors for ROP. BW was rescaled per 100 g increase (BW in grams divided by 100) to improve interpretability of regression coefficients. For binary and continuous variables, effect estimates are reported as odds ratios (OR) with 95% confidence intervals (CI) and coefficients (β) with 95% CI respectively. Multicollinearity in multivariable models for continuous variables was assessed using the variance inflation factor (VIF). When substantial collinearity was detected (VIF > 10), least absolute shrinkage and selection operator (LASSO) regression was applied for variable selection, and predictors with coefficients shrunk to zero were excluded from the final multivariable model. All tests were two-sided, and P < 0.05 was considered statistically significant.

## Results

Baseline characteristics, OCTA parameters, and visual acuity were compared between twins stratified into high and low BW groups. As shown in Table 1, sex distribution differed significantly between the groups (P = 0.047), with a higher proportion of males in the high BW group. The remaining baseline characteristics did not differ significantly (P > 0.05). OCTA parameters, visual acuity, and myopia prevalence were also not significantly different between the two cohorts.

**Table 1:** Baseline characteristics and examination results stratified by BW (high vs. low BW)

Parameters	High BW	Low BW	β (95%CI)	P value
No. of eyes/children	50/25	48/25	–	–
BW (g)	1179.76 ± 386.49	1038.00 ± 343.41	–	0.058
Age of examination (years)	5.88 ± 2.13	5.87 ± 2.36	–	0.788
Sex (male/female)	17/8	10/15	–	0.047
Mode of delivery (VD/CS)	15/10	15/10	–	1.000
ROP/Non-ROP	11/14	13/12	–	0.571
Myopia (yes/no)	5/20	6/19	–	0.733
FAZ area (mm2)	0.09 ± 0.11	0.09 ± 0.12	− 0.003 (− 0.049, 0.043)	0.907
FAZ boundary(mm)	0.99 ± 0.85	0.99 ± 0.96	0.006 (− 0.388, 0.401)	0.974
FAZ circularity	0.44 ± 0.33	0.38 ± 0.32	0.065 (− 0.082, 0.212)	0.385
Central foveal PD (mm^2^/mm^2^)	0.27 ± 0.07	0.26 ± 0.08	0.004 (− 0.034, 0.041)	0.849
Parafoveal PD (mm^2^/mm^2^)	0.39 ± 0.04	0.39 ± 0.05	− 0.001 (− 0.024, 0.022)	0.938
Foveal PD (mm^2^/mm^2^)	0.37 ± 0.04	0.37 ± 0.05	− 0.000 (− 0.023, 0.023)	0.979
Superior PD (mm^2^/mm^2^)	0.39 ± 0.05	0.39 ± 0.07	0.002 (− 0.026, 0.031)	0.874
Inferior PD (mm^2^/mm^2^)	0.38 ± 0.05	0.38 ± 0.05	− 0.003 (− 0.029, 0.022)	0.790
Temporal PD (mm^2^/mm^2^)	0.39 ± 0.04	0.40 ± 0.04	− 0.001 (− 0.017, 0.015)	0.904
Nasal PD (mm^2^/mm^2^)	0.39 ± 0.04	0.38 ± 0.07	0.013 (− 0.009, 0.035)	0.263
Central foveal VLD (mm/mm^2^)	14.85 ± 4.02	14.58 ± 4.39	0.284 (− 1.837, 2.405)	0.793
Parafoveal VLD (mm/mm^2^)	21.19 ± 2.32	21.19 ± 3.07	0.041 (− 1.373, 1.456)	0.954
Foveal VLD (mm/mm^2^)	20.47 ± 2.35	20.43 ± 3.02	0.074 (− 1.331, 1.480)	0.917
Superior VLD (mm/mm^2^)	21.31 ± 2.69	21.21 ± 3.83	0.137 (− 1.492, 1.766)	0.869
Inferior VLD (mm/mm^2^)	20.99 ± 2.71	21.04 ± 3.18	− 0.036 (− 1.506, 1.435)	0.962
Temporal VLD (mm/mm^2^)	21.62 ± 2.42	21.67 ± 2.53	− 0.034 (− 1.124, 1.056)	0.951
Nasal VLD (mm/mm^2^)	21.15 ± 2.21	20.55 ± 3.78	0.618 (− 0.710, 1.947)	0.362
CFT(μm)	122.74 ± 135.71	116.63 ± 134.22	2.098 (− 64.657, 68.853)	0.951
BCVA(LogMAR)	0.10 ± 0.13	0.11 ± 0.26	− 0.016 (− 0.127, 0.096)	0.785
SE (D)	1.03 ± 2.21	0.80 ± 2.02	0.350 (− 0.796, 1.495)	0.550

Mean differences and 95% CIs were estimated using GEE models accounting for inter-eye correlation.

*BW* birth weight, *VD* vaginal delivery, *CS* cesarean section, *ROP* Retinopathy of Prematurity, *FAZ* foveal avascular zone; *CFT* central foveal thickness, *SE* spherical equivalent, *BCVA* best-corrected visual acuity, *LogMAR* logarithm of the minimal angle of resolution, *PD* perfusion density, *VLD* vessel length density

Twins were further stratified by birth order (first-born vs second-born). No statistically significant differences were observed between first-born and second-born twins in any OCTA parameters or visual acuity outcomes (all P > 0.05; Table 2).

**Table 2:** Baseline characteristics and examination results stratified by birth order (first-born vs second-born)

Parameters	First-born	Second-born	β (95%CI)	P value
No. of eyes/children	49/25	49/25	–	–
BW (g)	1132.32 ± 399.51	1085.44 ± 342.08	–	0.399
Age of Examination (years)	5.88 ± 2.34	5.87 ± 2.16	–	0.867
Sex (male/female)	12/13	15/10	–	0.395
Mode of delivery (VD/CS)	15/10	15/10	–	1.000
ROP/Non-ROP	12/13	11/14	–	0.571
Myopia (yes/no)	5/20	6/19	–	0.733
FAZ area (mm^2^)	0.10 ± 0.11	0.07 ± 0.11	0.028 (− 0.017, 0.073)	0.218
FAZ Boundary(mm)	1.14 ± 0.90	0.84 ± 0.87	0.292 (− 0.092, 0.676)	0.136
FAZ Circularity	0.45 ± 0.31	0.37 ± 0.33	0.071 (− 0.075, 0.217)	0.342
Central foveal PD (mm^2^/mm^2^)	0.26 ± 0.08	0.27 ± 0.07	− 0.011 (− 0.048, 0.026)	0.553
Parafoveal PD (mm^2^/mm^2^)	0.39 ± 0.05	0.39 ± 0.04	− 0.001 (− 0.024, 0.022)	0.933
Foveal PD (mm^2^/mm^2^)	0.37 ± 0.05	0.37 ± 0.04	− 0.002 (− 0.025, 0.021)	0.851
Superior PD (mm^2^/mm^2^)	0.39 ± 0.06	0.39 ± 0.06	− 0.003 (− 0.032, 0.025)	0.813
Inferior PD (mm^2^/mm^2^)	0.38 ± 0.05	0.38 ± 0.05	− 0.005 (− 0.030, 0.021)	0.725
Temporal PD (mm^2^/mm^2^)	0.40 ± 0.03	0.39 ± 0.04	0.011 (− 0.005, 0.027)	0.191
Nasal PD (mm^2^/mm^2^)	0.38 ± 0.05	0.38 ± 0.06	0.001 (− 0.021, 0.024)	0.900
Central foveal VLD (mm/mm^2^)	14.40 ± 4.32	15.05 ± 4.07	− 0.567 (− 2.681, 1.547)	0.599
Parafoveal VLD (mm/mm^2^)	21.21 ± 2.74	21.17 ± 2.69	0.012 (− 1.401, 1.426)	0.986
Foveal VLD (mm/mm^2^)	20.43 ± 2.74	20.47 ± 2.66	− 0.056 (− 1.461, 1.348)	0.937
Superior VLD (mm/mm^2^)	21.14 ± 3.32	21.38 ± 3.27	− 0.227 (− 1.851, 1.397)	0.784
Inferior VLD (mm/mm^2^)	21.02 ± 2.97	21.01 ± 2.93	− 0.069 (− 1.538, 1.400)	0.927
Temporal VLD (mm/mm^2^)	21.85 ± 2.17	21.43 ± 2.73	0.382 (− 0.698, 1.462)	0.488
Nasal VLD (mm/mm^2^)	20.84 ± 3.10	20.88 ± 3.09	− 0.084 (− 1.411, 1.243)	0.901
CFT (μm)	136.51 ± 136.00	102.98 ± 131.87	33.762 (− 32.353, 99.878)	0.317
BCVA(LogMAR)	0.81 ± 0.20	0.85 ± 0.17	0.050 (− 0.056, 0.156)	0.356
SE (D)	1.27 ± 2.11	0.56 ± 2.08	0.635 (− 0.499, 1.768)	0.272

Mean differences and 95% CIs were estimated using GEE models accounting for inter-eye correlation.

*BW* birth weight, *VD* vaginal delivery, *CS* cesarean section, *ROP* Retinopathy of Prematurity; *FAZ* foveal avascular zone, *CFT* central foveal thickness, *SE* spherical equivalent, *BCVA* best-corrected visual acuity, *LogMAR* logarithm of the minimal angle of resolution, *PD* perfusion density, *VLD* vessel length density

**Fig. 2:**
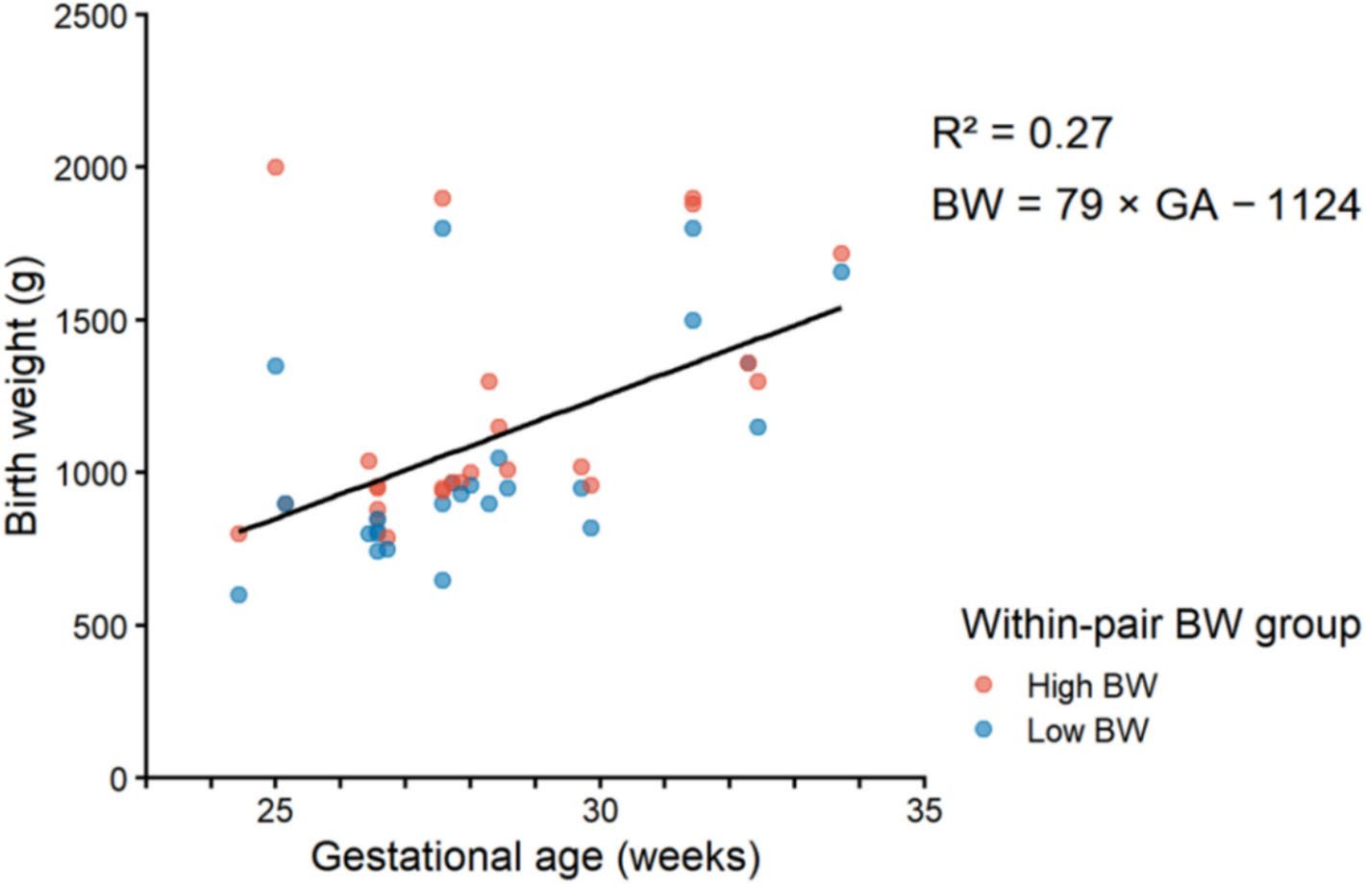
Relationship between GA and BW in preterm twin pairs. Each point represents one child; red indicates the co-twin in the within-pair High BW group and blue indicates the co-twin in the within-pair Low BW group. The solid line represents the least-squares linear fit across all infants (BW = 79 × GA − 1124; R^2^ = 0.27). *BW* birth weight, *GA* gestational age

As shown in Fig. 2, BW exhibited an overall upward trend with increasing GA among children from twin pairs categorized as within-pair high BW and low BW co-twins, and linear regression suggested a modest positive association (BW = 79 × GA − 1124; R^2^ = 0.27).

In the univariable and multivariable analysis of ROP risk factors in twins (Table 3), mode of delivery emerged as a significant factor (OR = 0.049, P = 0.005), suggesting that cesarean section was associated with a lower risk of ROP compared to vaginal delivery. No significant associations were observed between ROP and sex, birth order, GA, or BW (all P > 0.05).

**Table 3:** Univariable and multivariable analysis of ROP risk factors in twins by the GEE model

Parameters	Univariable OR (95% CI)	P value	Multivariable OR (95% CI)	P value
FAZ area (mm^2^)	1.000 (1.000, 1.000)	0.055	1.047 (0.958, 1.144)	0.311
Birth order (Second-born/first-born)	0.852 (0.281, 2.585)	0.777	0.938 (0.246, 3.576)	0.925
Mode of delivery (CS/VD)	0.248 (0.074, 0.832)	0.024	0.049 (0.006, 0.407)	0.005
GA (weeks)	1.119 (0.879, 1.423)	0.362	1.431 (0.967, 2.117)	0.073
BW (g)	1.001 (0.999, 1.002)	0.440	1.001 (1.000, 1.003)	0.107
Sex (Male/Female)	1.175 (0.386, 3.576)	0.777	0.557 (0.118, 2.628)	0.460

ROP was coded as 0 = No, 1 = Yes; Birth order was coded as 0 = first-born, 1 = second-born; Mode of delivery; Mode of delivery was coded as 0 = vaginal delivery, 1 = cesarean section.

*GA* gestational age, *VD* vaginal delivery, *CS* cesarean section, *BW* birth weight, *FAZ* foveal avascular zone, *OR* odds ratio, *CI *confidence interval

We explored the impact of various risk factors on BCVA (LogMAR) through univariable and multivariable analyses (Table 4). The univariable analysis revealed that higher foveal PD (β = − 0.826, P = 0.046) and higher GA (β = − 0.015, P = 0.014) were significantly associated with better BCVA. Other OCTA parameters showed no statistically significant associations with BCVA in the univariable models (all P > 0.05). In the multivariable analyses, GA remained independently associated with BCVA (β = − 0.013, P = 0.030), whereas the association for foveal PD was attenuated and no longer statistically significant (β = − 0.621, P = 0.128). These results suggest that changes in BCVA are likely influenced by the combined effect of multiple factors rather than any single parameter, but they highlight the potential importance of FAZ-related morphology and retinal perfusion levels in visual acuity outcomes.

**Table 4:** Univariable and multivariable analysis of factors associated with BCVA (LogMAR) by the GEE model

Parameters	Univariable		Multivariable	
	β (95%CI)	P value	β (95%CI)	P value
FAZ area (mm^2^)	− 0.592 (− 1.225, 0.042)	0.067		
FAZ boundary(mm)	− 0.070 (− 0.144, 0.004)	0.066		
FAZ circularity	− 0.206 (− 0.421, 0.009)	0.061		
Foveal PD (mm^2^/mm^2^)	− 0.826 (− 1.636, − 0.015)	0.046	− 0.621 (− 1.421, 0.179)	0.128
Parafoveal PD (mm^2^/mm^2^)	− 1.078 (− 2.175, 0.020)	0.054		
Central foveal PD (mm^2^/mm^2^)	0.515 (− 0.226, 1.256)	0.173		
Foveal VLD (mm/mm^2^)	− 0.023 (− 0.048, 0.002)	0.068		
Parafoveal VLD (mm/mm^2^)	− 0.027 (− 0.055, 0.002)	0.071		
Central foveal VLD (mm/mm^2^)	0.005 (− 0.006, 0.016)	0.337		
CFT (μm)	0.0004 (− 0.0001, 0.0008)	0.109		
BW (per 100 g)	0.003 (− 0.006, 0.012)	0.552		
GA(weeks)	− 0.015 (− 0.027, − 0.003)	0.014	− 0.013 (− 0.025, − 0.001)	0.030
Birth order	− 0.050 (− 0.156, 0.056)	0.356		

*BW* birth weight, *VD* vaginal delivery, *CS* cesarean section, *ROP* retinopathy of prematurity, *FAZ* foveal avascular zone, *CFT* central foveal thickness, SE spherical equivalent, BCVA best-corrected visual acuity, *logMAR* logarithm of the minimum angle of resolution, *PD* perfusion density, *VLD* vessel length density

We further examined factors associated with FAZ area using univariable and multivariable analyses by the GEE and LASSO regression models respectively (Table 5). Univariable analysis revealed that longer FAZ boundary (β = 0.116, P < 0.001), higher FAZ circularity (β = 0.201, P < 0.001), higher parafoveal PD (β = 0.590, P < 0.001), higher foveal VLD (β = 0.006, P = 0.027), higher parafoveal VLD (β = 0.011, P < 0.001), and higher GA (β = 0.013, P = 0.005) were significantly associated with larger FAZ area. Additionally, higher central foveal PD (β = − 0.728, P < 0.001), central foveal VLD (β = − 0.012, P < 0.001), and higher CFT (β = − 0.0002, P = 0.019) were significantly associated with smaller FAZ area.

**Table 5:** Univariable and multivariable analysis of factors associated with FAZ area

Parameters	Univariable		Multivariable	
	β (95%CI)	P value	β (95%CI)	P value
FAZ boundary (mm)	0.116 (0.091, 0.140)	< 0.001	0.133 (0.107, 0.158)	< 0.001
FAZ circularity	0.201 (0.147, 0.254)	< 0.001	−0.113 (− 0.166, − 0.059)	< 0.001
Foveal PD (mm^2^/mm^2^)	0.308 (− 0.019, 0.635)	0.065		
Parafoveal PD (mm^2^/mm^2^)	0.590 (0.363, 0.816)	< 0.001	–	–
Central foveal PD (mm^2^/mm^2^)	− 0.728 (− 1.087, − 0.368)	< 0.001	–	–
Foveal VLD (mm/mm^2^)	0.006 (0.001, 0.012)	0.027	–	–
Parafoveal VLD (mm/mm^2^)	0.011 (0.006, 0.015)	< 0.001	–	–
Central foveal VLD (mm/mm^2^)	− 0.012 (− 0.019, − 0.006)	< 0.001	− 0.004 (− 0.005, − 0.002)	< 0.001
CFT (μm)	− 0.0002 (− 0.0003, − 0.0000)	0.019	–	–
BW (per 100 g)	0.001 (− 0.004, 0.005)	0.732		
GA (weeks)	0.013 (0.004, 0.023)	0.005	0.004 (0.002, 0.006)	< 0.001
Birth order	− 0.028 (− 0.073, 0.017)	0.218		
Age of examination (years)	− 0.003 (− 0.012, 0.005)	0.449		

Univariable analyses were performed using GEE model. To address multicollinearity, candidate predictors were selected using least absolute shrinkage and selection operator (LASSO) regression; variables with coefficients shrunk to zero were excluded from the final multivariable model.

*GEE* generalized estimating equation, *BW* birth weight, VD vaginal delivery, *CS* cesarean section, *ROP* retinopathy of prematurity, *FAZ* foveal avascular zone, *CFT* central foveal thickness, *SE* spherical equivalent, *BCVA* best-corrected visual acuity, *logMAR* logarithm of the minimum angle of resolution, *PD* perfusion density, *VLD* vessel length density

Due to multicollinearity (VIF > 10), LASSO regression was used for variable selection, and predictors with coefficients shrunk to zero were excluded from the final multivariable model. In the LASSO-selected multivariable model, GA (β = 0.004, P < 0.001) and FAZ boundary (β = 0.133, P < 0.001) remained positively associated with FAZ area, whereas FAZ circularity (β = − 0.113, P < 0.001) and central foveal VLD (β = − 0.004, P < 0.001) were independently negatively associated with FAZ area.

## Discussion

This study employed OCTA to evaluate the macular microvascular parameters (FAZ, PD, VLD), CFT and visual outcomes (BCVA and SE) in preterm twins aged 3 to 12 years. In this cohort, 24 of 50 children (48%) had a documented history of ROP, higher than the ROP prevalence of 31.9% reported for general preterm populations [28]. When stratified by BW or birth order, no significant differences were observed in macular microvascular metrics and BCVA. Cesarean section was associated with a lower risk of ROP compared to vaginal delivery. Moreover, GA showed a positive association with FAZ area and BCVA in multivariable analyses, highlighting GA as an important perinatal correlate of long-term macular development and visual function in preterm twins.

Stratification by BW revealed no significant differences between the high BW and low BW groups in FAZ, PD, VLD, CFT or BCVA. This suggests that, within preterm twins, BW alone may not be the primary determinant of long-term retinal microvascular morphology or ultimate visual outcomes, consistent with broader preterm cohorts in which the influence of BW on certain retinal metrics diminishes or disappears after adjusting for GA [29].

Similarly, no significant differences in OCTA parameters or visual outcomes were observed between first-born and second-born twins. This finding differs from the report by Yau et al [30] in Chinese twin cohorts, who found that first-born twins had a higher risk of complications, including ROP. The discrepancy may partly reflect differences in study endpoints, sample characteristics, and contemporary perinatal management protocols. However, a more granular comparison was not feasible. This is because detailed perinatal records, including oxygen exposure duration and Apgar scores, were not consistently retrievable. Future prospective studies with systematic perinatal data capture are warranted.

Birth order has been investigated as an important perinatal factor in twin research because second-born twins have a higher risk of delivery-related perinatal death and intrapartum hypoxia compared with first-born twins [31–33]. From an ophthalmic perspective, such differences in perinatal hypoxic burden could plausibly influence retinal and macular development. Although no birth-order–related differences in ocular structure or function were found in this study, it remains appropriate to treat birth order as a predefined perinatal variable in the present analyses, given its known association with perinatal risk.

This study also extended the association of cesarean delivery as a protective factor against ROP occurrence to the preterm twin population, consistent with previous studies [34, 35]. The underlying mechanism may involve distinct peripartum stress responses: vaginal delivery exposes infants to transient hypoxia–reoxygenation cycles and inflammatory mediators, which can exacerbate immature antioxidant defenses and precipitate retinal vascular dysregulation in preterm neonates [36, 37]. Although suggestive, this finding is preliminary and requires confirmation in prospective studies that rigorously control for additional perinatal confounders, such as the duration of labor and the indications for emergency cesarean delivery.

Sex was not identified as a significant risk factor for ROP in this twin cohort, despite differences in sex distribution between the within-pair high BW and low BW groups. Similar findings have been reported in several clinical studies in which male sex did not emerge as an independent predictor of ROP [23, 38, 39], whereas other large cohort and meta-analytic investigations have suggested that male sex may modestly increase the risk of ROP [40, 41]. These inconsistent findings suggest that the role of sex in ROP pathogenesis remains incompletely understood. Future large-scale, prospective studies with adequate statistical power are needed to determine whether sex represents an independent risk factor and to quantify any sex-specific differences in ROP susceptibility.

In analyses of BCVA, higher GA was consistently associated with better BCVA. Foveal PD was associated with better BCVA in univariable models; however, this association was attenuated after adjustment, suggesting that macular perfusion metrics may share variance with GA rather than acting as independent determinants. Other OCTA parameters and CFT showed no significant associations with BCVA. This is consistent with prior reports observing no significant association between FAZ area and visual acuity in preterm children [42]. Another study confirmed that increased CFT in preterm children does not significantly correlate with visual function [43]. The limited sample size in this study may have reduced statistical power. Consequently, larger prospective studies are required to disentangle the complex interplay of these factors and determine their independent contributions to visual acuity.

This study demonstrated a significant positive association between GA and FAZ area, identifying GA as an independent perinatal determinant of FAZ morphology. This finding aligns with previous developmental arrest hypothesis proposed by Mintz-Hittner et al [29] and is consistent with multiple prior studies [5, 25, 26, 44]. Furthermore, it provides direct anatomical support for the centrifugal remodeling theory of Henkind et al [45], which posits that the FAZ forms postnatally through capillary displacement from an initially vascularized fovea. Building on our team’s prior findings regarding macular microvascular differences in ROP patients treated with anti-vascular endothelial growth factor (anti-VEGF) injection versus laser therapy [44], the present study further validates the use of OCTA in preterm twins and reinforces GA as a robust predictor of long-term macular microvascular morphology.

Multivariable LASSO regression analysis identified that a longer FAZ boundary was independently associated with a larger FAZ area, whereas higher FAZ circularity was associated with a smaller FAZ area. Notably, central foveal VLD showed a significant independent negative association with FAZ area (P < 0.001), indicating that higher central foveal VLD corresponds to a smaller FAZ. This finding is consistent with previous studies reporting a strong negative correlation between FAZ area and both PD and VLD in the 1-mm central area [44, 46]. Cross-study comparisons of FAZ and perfusion parameters should be interpreted cautiously because OCTA measurements can vary by device. Cirrus and AngioVue show systematic FAZ differences and limited agreement, which may contribute to inconsistent findings regarding the relationship between FAZ and perfusion parameters findings [47]. Moreover, pediatric normative studies report superficial FAZ area estimates of approximately 0.26–0.47 mm^2^ across devices and protocols, and age-related variability may further broaden the distribution in children [48]. Taken together, these findings suggest that inconsistent associations between the FAZ and perfusion parameters across studies may be driven by both platform-related measurement variability and pediatric biological heterogeneity. Future multicenter studies with standardized OCTA protocols are warranted to validate and refine these associations in pediatric populations.

Although CFT was associated with FAZ area in the univariable analysis, this association did not remain significant after multivariable adjustment, indicating that its effect on FAZ morphology may be confounded or mediated by GA or other OCTA parameters. This is noteworthy given that previous studies have consistently reported that preterm children exhibit increased CFT compared to full-term peers [6, 49, 50], which is attributed to incomplete centrifugal migration of inner retinal neurons resulting in their persistence at the foveal center [51].

This study has several limitations. First, the sample size was modest, and the limited number of outcome events may have reduced statistical power and the stability of multivariable models. Second, this single-center observational study conducted at a tertiary ophthalmic specialty hospital cannot establish causality and may have limited generalizability. Third, key perinatal and neonatal variables, such as oxygen exposure and Apgar scores, were not consistently available, which may introduce residual confounding. In addition, birth-order analyses were restricted to within-pair comparisons, and twin zygosity data were unavailable, precluding assessment of genetic influences; larger multicenter prospective studies are warranted.

## Conclusion

Birth order and BW in preterm twins did not affect ROP incidence, long-term macular structure, microvascular parameters, or visual outcomes. Lower GA was associated with smaller FAZ area and poorer visual acuity in preterm twins. OCTA offers a useful, noninvasive approach for assessing long-term foveal characteristics in preterm populations.

## Data Availability

No datasets were generated or analysed during the current study.

## Abbreviations

ROP: Retinopathy of prematurity
OCTA: Optical coherence tomography angiography
BW: Birth weight
GA: Gestational age
BCVA: Best-corrected visual acuity
CFT: Central foveal thickness
FAZ: Foveal avascular zone
LogMAR: Logarithm of the minimal angle of resolution
SE: Spherical equivalent
PD: Perfusion density
VLD: Vessel length density
